# Insect Meal as a Dietary Protein Source for Pheasant Quails: Performance, Carcass Traits, Amino Acid Profile and Mineral Contents in Muscles

**DOI:** 10.3390/ani14202992

**Published:** 2024-10-17

**Authors:** Marian Flis, Piotr Czyżowski, Grzegorz Rytlewski, Eugeniusz R. Grela

**Affiliations:** 1 Department of Animal Ethology and Wildlife Management, Animal Sciences and Bioeconomy, University of Life Sciences in Lublin, 20-950 Lublin, Poland; marian.flis@up.lublin.pl; 2 Polish Hunting Association Gdańsk District Board, 80-286 Gdańsk, Poland; goigr@wp.pl; 3 Institute of Animal Nutrition and Bromatology, Animal Sciences and Bioeconomy, University of Life Sciences in Lublin, 20-950 Lublin, Poland; eugeniusz.grela@up.lublin.pl

**Keywords:** pheasant, insect meal, muscles, amino acids, minerals

## Abstract

The aim of the study was to determine the effects of replacing soybean meal with insect meal on the body weight and the chemical composition of selected muscle groups of common pheasant females and males, including the mineral composition and the amino acid profile of the thigh and breast muscles. The study was conducted on three feeding groups, namely one control and two experimental groups. In the control group, plant feed components were used, which are commonly used to feed pheasants in confined breeding facilities. In the experimental groups, 100 g (group II) and 200 g (group III) portions of insect meal were introduced instead of the plant-protein components. The experiment used a preparation of insect larvae (*Hermetia illucens*) containing approximately 52% crude protein. The pheasant diet supplementation applied contributed to an increase in the proportion of muscles in the carcasses, with the highest effectiveness obtained for a 20% addition of insect meal. Lower and significant differences were noted in the feed conversion by birds from the experimental groups, as compared to the control group. The chemical composition of the birds’ muscles also changed. The experimental groups exhibited higher protein and fat contents and a lower water content. No significant changes in the amino acid profile or the mineral composition of the muscles were noted. The few exceptions concerned the methionine levels in both muscle groups and the isoleucine levels in the breast muscles. In most cases, the mineral composition did not vary significantly (*p* < 0.05). When supplementing the diet of breeding pheasants for improving meatiness, a 20% addition of insect meal is recommended, which affects the production effect of this trait while reducing feed consumption and maintaining the fatty acid profile.

## Simple Summary

In recent years, confined pheasant farms have been gaining popularity in both stocking and the production of meat and eggs. When assessing the nutritional value of meat products, the focus is on the nutrient content, including the mineral content and the amino acid profile. The production performance, e.g., body weight gains, feed conversion, and the proportion of meat in the carcasses, are also not insignificant. At the same time, taking into account the feeding behaviour of pheasants, especially during the rearing period under farm conditions, varied dietary components are used, including meals of animal origin and mineral-and-vitamin supplements. The authors hypothesised that the introduction of insect meal to the diet, instead of plant protein feeds, would have an effect on the production performance and the nutritional components of the muscles. The study results obtained show that the addition of insect meal contributed to an improvement in the meat yield and the levels of certain minerals without significantly affecting the amino acid profile. A 20% addition of meal from the larvae of insects *Hermetia illucens* proved to be most beneficial.

## 1. Introduction

In Europe, the first records of common pheasants *Phasianus colchicus* kept in confined breeding facilities date back to the 16th century and come from the Silesian region. With the development of breeding and the supply of birds, they have rather rapidly become a species used for restocking natural habitats, and due to the rapid growth of the population, pheasants have become game animals. Thus, they have become a hunter-managed and game species, which continues to the present day [1,2,3]. The usable value, particularly the nutritional, dietary, and even health-promoting qualities of pheasant meat, was also quickly recognised. Hence, in addition to keeping the birds in stocking farms to reinforce local populations and establish new ones, peasant fattening farms have emerged [4,5,6,7]. When the birds are kept for slaughter, the important breeding factors include the weight of the birds to be slaughtered, their slaughter yield, and the physicochemical properties of the individual muscles used for culinary purposes. In order to improve the culinary value of meat and to intensify fattening, new solutions are being sought to optimise weight gain, feed conversion and meat quality, both for immediate consumption and for storage [8,9,10,11]. The work in this field is aimed at optimising the protein and energy levels as well as the sources of minerals and vitamins by means of appropriate supplementation of feed mixtures used in pheasant feeding [12,13,14]. In recent years, there has been a trend towards optimising the feeding of farm birds to make it more similar to natural feeding, where the diet is based on the consumption of different invertebrate species and forms. These solutions are based on the addition of larvae or insect meal to the feed, with this supplementation having a possible impact on body weight gains, feed conversion, and the quality of meat and eggs obtained as a raw material for consumption [15,16].

So far, black soldier fly, mealworm, housefly, Grasshopper (Orthoptera), silkworm, and earthworm have been primarily used in poultry feeding, especially for broilers and laying hens [17]. The study’s results [18] indicate that adding insect meals to the diet can improve growth and feed conversion efficiency, suggesting their usefulness in poultry nutrition. In addition, using insect meals in poultry diets contributes to reduced environmental pressure (due to the use of plant waste in insect farming) compared to other protein sources [19]. A study showed that the black soldier flies’ larvae digest organic waste in one week, causing CO_2_ emissions of 28% compared to bacterial decomposition, which took 45 days and CO_2_ emissions of 50% [20]. It should also be noted that the larvae of the black soldier fly are not disease vectors; they have a high protein content (37–63%), a favourable amino acid profile, and a higher fat content [21]. The proportion of insect meal in animal nutrition can modify the quantity and quality of animal products [22]. The results of researchers who analysed the pectoral muscles of Japanese quails and broiler chickens indicated that the proportion of saturated fatty acids was higher in the muscles of birds fed with a meal from larvae (*Hermetia illucens*) [23,24]. Studies on the dietary supplementation of pheasants with insect meal are few and can be described as pioneering [16]. According to the European regulations approved in March 2020 on the so-called Green Deal regarding climate independence and environmental sustainability, insect farming waste as a feed additive is allowed within the recycling framework. This is mainly due to its high protein content, which can be used as a dietary supplement for poultry and other species by replacing post-extraction soybean meal. In addition, the production process is relatively simple, and most importantly, they release less ammonia and liquid gases than traditional livestock, while using waste materials that would have to be recycled as substrates. Insect meals have high nutritional values, including up to 70% crude protein, and are a valuable source of minerals, including potassium, iron, phosphorus, copper, zinc, and B vitamins. They lack carbohydrates, and are classified as gluten-free [25].

The aim of the study was to determine the effect of replacing soybean meal with insect meal on the body weight, slaughter performance, and the chemical composition of selected muscle groups of common pheasant females and males, including the mineral composition and the amino acid profile.

## 2. Materials and Methods

### 2.1. Feed

The diet for the control group comprised plant fodder components that are used in the feeding of pheasants (Table 1). In the experimental groups, 100 g (group II) or 200 g (group III) portions of insect meal (*Hermetia illucens)* were introduced instead of vegetable protein components (soybean meal—group II and soybean meal, garden pea and partly sunflower meal—group III). Insect meal (IM) by HiProMine S.A. (Poznań, Poland), containing approximately 52% of crude protein, was used in the experiment (Table 2). All the feed components were mixed and pelleted (0.5 mm diameter) at 60 °C.

### 2.2. Animals

The research was carried out on experimental groups of cock and hen game pheasants bred for fattening. The experiment was conducted on a farm of pheasants kept in aviaries in Wierzchowiny village (Radzyn district, Poland). The location of the farm is (latitude 51.611514411746676, geographic longitude 22.69260406494141).

Three groups with different nutrition were established, as follows: one control group and two experimental groups (Table 1). The pheasants (eight cocks and eight hens) were treated together as a study group and were kept in flight pens measuring 8.5 m long × 5.0 m wide × 3.5 m high. The experiment was carried out on four-week-old pheasants, who were fed ad libitum with mixtures intended to fatten them during the rearing period (Table 2) until they reached the age of 28 days. After this period, a grower (5–8 weeks of life) and finisher (9–16 weeks) feed mix was used, with different amounts of insect meal for individual feeding groups. For the duration of the study, feeding was at will, and the birds had free access to water. In the experiment, they were weighed after week 4, and then after weeks 8 and 16. Daily feed intake was monitored by weighing the mixture into feeders and recording the weight of any unconsumed feed. At the end of the experiment, when all pheasants were 16 weeks old, they were weighed, decapitated, and re-weighed after exsanguination. Another weighing was carried out after the pheasants had been plucked and eviscerated. Two groups of meat were prepared from carcasses, namely breast and thigh. The muscles were stored in a refrigerator at 4 °C until chemical analyses were carried out.

### 2.3. Analytical Procedures

The chemical composition (dry matter, crude protein, crude fibre, crude fat, crude ash and amino acids) of the diets and tissue (except crude fibre) samples were analysed according to AOAC procedures (2010) [26]. The mineral content was determined by the flame atomic absorption spectroscopy (FAAS) method. Mineralisation was carried out using a Mars Xpress apparatus by CEM (Stanford, CA, USA) in Teflon dishes. The process was conducted using nitric acid (V) in the amount of 4 cm^3^ per 0.5 g of sample (nitric acid (V) Suprapur, manufactured by Merck (Darmstadt, Germany)). The process parameters were as follows: magnetron power of 800 W; temperature rise for 25 min; temperature of 210 °C. The temperature was maintained for 15 min. After mineralisation, the samples were quantitatively transferred into 50 cm^3^ volumetric flasks and diluted with demineralised water to the mark. For the analyses, a SpektrAA 280FS flame atomic absorption spectrometer manufactured by Varian was utilised, with an SPS-3 autosampler and a SIPS dilutor (Melbourne, Australia). As a dilutor is used for the calibration curve, only the stock solution, taken and diluted by the apparatus, is prepared. The individual samples are poured into test tubes and placed in the autosampler, from which they are automatically dispensed to the apparatus. Standards manufactured by CPA Chem (Bogomilovo, Stara Zagora, Bulgaria) and distributed by LGC Standards (concentration of 1000 mg/dm^3^, matrix: 2% nitric acid (V)) were certified in accordance with standard ISO 17034 [27].

The amino acid content was determined in accordance with [28]. The sample was hydrolysed at 110 °C for 20 h in 6N HCl using an Ingos HBO 16 hydrolyser (Prague, Czech Republic). After cooling, the solution containing the sample was filtered through a G-5 funnel. The hydrolysate was then evaporated on an RVO 400 vacuum evacuator (MERAZET S.A. Poznań, Poland). The dry residue from the vacuum flask was dissolved in a citrate buffer with a pH of 2.2. The prepared sample, after being filtered through a syringe filter, was dispensed onto the column of an AAA 400 Ingos amino acid analyser (Prague, Czech Republic). The hydrolysis of proteins for the separation of amino acids was carried out in accordance with [29]. Cysteine was oxidised to cysteic acid, and methionine was oxidised to methionine sulfone using performic acid. The mixture was then poured over with 48% HBr and thickened on an RVO 400 vacuum evaporator (MERAZET S.A. Poznań, Poland) and then poured over with 6N HCl and transferred into the HBO 16 Ingos hydrolyser extraction thimble. The hydrolysis was carried out as previously described.

The determination of tryptophan was carried out in accordance with [30]. The sample was hydrolysed in a Ba(OH)_2_ solution using an HBO 16 Ingos hydrolyser. The sample was then acidified with 6N HCl, and a Na_2_SO_4_ solution was added to precipitate barium ions. The content was transferred to centrifuge test tubes, and the precipitated BaSO_4_ was centrifuged for 15 min at 3000× *g* using a ROTOFIX 32A Hettich centrifuge (Andreas Hettich GmbH & Co. KG, Tuttlingen, Germany). The supernatant, after being filtered through a syringe filter, was dispensed onto the AAA 400 amino acid analyser. Amino acids were determined using an AAA 400 Ingos amino acid analyser (PragueCzech, Republic). Amino acids were separated by the ion-exchange chromatography method. A column with dimensions of 0.37 cm × 45 cm was filled with an ion exchanger in the form of a resin. Ostion LG ANB was used for the hydrolysates. This is a strong cation exchanger with an average grain size of approximately 12 µm in the form of Na. The column temperatures were 60 °C and 74 °C. The apparatus detects amino acids by derivatisation with ninhydrin (i.e., a reagent for detection). Amino acids are identified using a photometric detector at a wavelength of 570 nm for all amino acids (440 nm only for proline). Four buffers were used for the separation: 1—pH 2.6; 2—pH 3.0; 3—pH 4.25; 4—pH 7.9. After the amino acid separation, the column was regenerated with 0.2 N NaOH. The mobile phase involved the individual buffers. Pump 1 was ninhydrin, and the flow rate was 0.3 mL/min, while pump 2 was water, and the flow rate itself was 0.25 mL/min.

### 2.4. Statistical Analysis

A two-factor analysis of variance was performed to determine the differences between the average values of the traits analysed by feeding group and sex. In order to determine the differences between the muscles, an ANOVA single-factor analysis was performed. The Newman–Keuls test was used in Statistica 10.0 (StatSoft, Tulsa, OK, USA) to verify the significance of the differences between the mean values. The effect of nutrition, sex, and muscle type was found to be notable at the significance level (*p* ≤ 0.05).

## 3. Results

The weight of the birds on day 29 was very similar, with a pronounced sexual dimorphism in favour of males (Table 3). Diversified feeding, including insect meal, contributed to an increase in the body weight at the end of fattening by 68 g in group II and by 137 g in group III, which was close to the significance level (*p* = 0.078). Feed consumption per gram of gain was definitely the lowest in the feeding group, in which 20% of the dietary supplementation with insect meals was applied. The average values of this characteristic differed significantly between the feeding groups and the control group (*p* ≤ 0.05). There were distinct differences in the body weight of pheasant males and females and significant differences in feed conversion (Table 3). In addition, significant differences between the feeding groups and the pheasants’ sex were noted. The chemical composition of both the breast muscles and thigh muscles of the birds fed feed with the addition of insect meal showed higher protein and fat contents and a lower water content (Table 4). These differences were not statistically significant. Moreover, differences were noted in the protein and fat contents within the birds’ sex groups. However, a statistically significant difference was only noted for the fat content of the breast muscles of hens and cocks.

There was very low diversity in the amino acid levels in the individual muscle types with regard to sex and feeding groups (Table 5). High and statistically significant differences in the methionine (Met) levels between feeding groups were noted in both breast and thigh muscles. Statistically significant differences between the individual feeding groups were also noted in the isoleucine (Ile) and leucine (Leu) levels, but only within the breast muscle region. Significant differences between males and females only occurred in the glutamic acid (Glu) levels. On the other hand, no significant differences were observed in the levels of the amino acids under study between the feeding groups and the birds’ sex.

An assessment of the macro- and micronutrient content revealed that the dietary supplementation did not have a significant effect on the contents of individual elements in the birds’ muscles (Table 6). A 20% addition of insect meal contributed significantly to increased potassium and copper contents of the birds’ breast muscles, as well as iron and zinc contents of the thigh muscles. The contents of other elements under assessment did not differ significantly between the feeding groups or within the analysed groups of pheasant muscles. At the same time, dietary supplementation had a significant impact on the macro- and micronutrient contents in the individual muscle groups between the hens and the cocks. In the hens, a higher and statistically significant content of all the macro-nutrients was noted in both the thigh and breast muscles, with the exception of sodium, the content of which was also higher and close to the significance level. The proportion of micronutrients in the muscles varied, and significantly higher contents in both the breast and thigh muscles were demonstrated for iron and zinc. No significant interactions in the macro- and micronutrient contents were noted between the groups and the birds’ sex.

## 4. Discussion

In recent years, new solutions for the supplementation of animal diets have been sought in order to intensify production by increasing the performance indicators of a particular production line. These trends have also concerned the confined breeding of wild animals. As regards pheasants, solutions are being sought to influence egg yield and the chemical composition of eggs, the weight of muscles and their chemical composition, and the anatomical and morphological characteristics [31,32]. Studies on the replacement of inorganic sources with organic forms of minerals in the diets of farmed poultry showed better bioavailability and, thus, their increased deposition in the birds’ tissues [33,34,35]. A study on the partial replacement of Ca, Fe, Zn and Cu salts with glycine chelates showed a higher final weight of pheasant hens and an increase in many mechanical parameters of the bones [13]. In poultry production, the use of L-carnitine is quite common, as it contributes to the stimulation of growth, and thus body weight gains, to the strengthening of the immune system, and to a reduction in the abdominal fat in boilers [36,37,38].

In the search for new nutritional solutions, insect larvae or meals from these larvae have been increasingly utilised in replacing plant protein with animal protein in recent years. Insect meal is considered one of the most suitable alternatives to plant protein in the feeding of monogastric animals [39]. Insect meal has many advantages over insects fed whole. Some insects cannot be used directly because they secrete toxins or harmful minerals [40]. Therefore, they should be processed to make them safe for poultry diets. Live insects may be difficult to handle, but they also have the potential to act as vectors in the transmission of bacterial and viral diseases [41].

The authors’ study on pheasants demonstrated that the addition of larval meal (*Hermetia illucens*) to the pheasants’ diet had a positive effect on both the number of eggs laid and the parameters of the birds’ hatching rate [16]. The same study conducted on pheasants revealed that dietary supplementation using insect meal (*Hermetia illucens*) had an effect on numerous anatomical and morphological traits of pheasants, with the most significant being an increase in the weight of the leg muscles, which is of great importance for breeding for the purpose of restocking, because of the specific behaviour of these birds [7]. Insect meal contains a higher amount of essential amino acids, as compared to conventional feeds [42,43,44]. Thus, the results of our research confirm that adding insect meal to the feed at a level of 20% is optimal for increasing production effects in meat utilisation by increasing the proportion of muscle in carcasses. Such dietary supplementation also contributes to improving the chemical composition as well as the amino acid profile. It should also be emphasised that the implementation of pheasants’ diets proposed by us, through the use of insect meal, has the effect of bringing the composition of feed under farming conditions closer to that of wild pheasants, in which the diet includes not only cereal grains, but also protein of animal origin in the form of invertebrates [45]. At the same time, the results obtained confirm that the use of insects in the diet of poultry, including wild birds, as alternative sources of protein provide better production rates and a higher quality of final products in comparison with fish meal or soybean meal [46,47,48].

The results of studies on the addition of insect meals to bird diets are inconsistent due to the differences in the compositions of meals and the bird species or breed [49,50].

A study on the effects of insect larval meal on the characteristics of the meat of Japanese quails (*Coturnix japonica*) showed an increase in the feed conversion ratio but a decrease in the carcass and breast weight, whereas the fatty acid composition did not change [51].

Cullere et al. [52,53] found that the proportion of saturated and monounsaturated fatty acids increased with an increase in the content of protein from black soldier fly (*Hermetia illucens*) in the diet, to the detriment of the polyunsaturated fatty acid fraction, thus reducing the healthiness of breast meat. The contents of aspartic acid, glutamic acid, alanine, serine, tyrosine, and threonine increased. The meat of quails fed the feed with the highest proportion of black soldier fly larvae meal showed the highest Ca and P contents. These authors believe that black soldier fly larvae meal (*Hermetia illucens*) is a promising source of insect protein for quails, with its only disadvantage being the deterioration of the fatty acid profile. Another study on the addition of black soldier fly larvae *Hermetia illucens* [54] found that the protein in the larvae was rich in lysine and methionine but deficient in histidine and leucine.

The addition of insect meal to the diet of farmed pheasants adds variety and makes it more similar to a natural diet. As shown by a study by Whiteside et al. [55], pheasants fed a more naturalistic diet, after being released into the wild, are more successful at finding and assimilating natural food thanks to the development of proper intestinal morphology.

The use of insect meal in the diet of pheasants is not associated with significant restrictions. Although the replacement of post-extraction soybean meal with insect meal is associated with a slightly different amino acid composition and higher fat content, under natural conditions, the insect protein is the primary component of the diet of these birds, especially young developing individuals. Thus, it is worth exploring the possibility of using defatted insect meal as a substitute for post-extraction soybean meal.

## 5. Conclusions

The use of insect meal (*Hermetia illucens*) instead of plant protein components in pheasant quail diets contributed to an improvement in the production performance and the proportion of muscles in the carcasses, with an addition of 20% of the mixture being the most effective. A variation in the birds’ body weight and significant differences in feed conversion were observed. In the feeding groups with dietary supplementation, higher protein and fat contents and lower water content were observed. No significant changes were noted in the pheasant muscle protein amino acid profile or the mineral composition of the muscles. The exceptions included the methionine levels in both muscle groups, as well as the isoleucine and leucine levels within the breast muscle region. On the other hand, significant differences between the sex groups were noted in the glutamic acid levels and only in the thigh muscles. The mineral composition of the individual muscle groups also did not differ significantly between the feeding groups. The exceptions included the potassium and copper contents of the breast muscles and the iron and zinc contents of the thigh muscles. Significant differences were found in the contents of most minerals between the sex groups, with the exception of copper and manganese. No significant differences were noted between the feeding groups and the birds’ sex.

## Figures and Tables

**Table 1:** Ingredient composition (g/kg) of the pheasant diet.

Diet	0–4 Weeks	5–8 Weeks			9–16 Weeks		
		Control	10% IM	20% IM	Control	10% IM	20% IM
Corn	330.3	314.2	314.2	314.2	316.6	316.6	316.6
Wheat	130	160	180	200	290	310	320
Soybean meal	300	280	160	40	140	20	0
Garden pea	20	50	50	50	50	50	0
Linseed	40	40	40	40	40	40	40
Sunflower meal	50	80	80	80	80	80	40
Fish meal	70	10	10	10	0	0	0
Insect meal	0	0	100	200	0	100	200
Sorghum	20	30	30	30	50	50	50
Dicalcium phosphate	12	13	13	13	13	13	13
Calcium carbonate	15	12	12	12	10	10	10
Sodium chloride	5	5	5	5	5	5	5
Mineral–vitamin premix ^1^	2.5	2.5	2.5	2.5	2.5	2.5	2.5
DL-methionine	1.2	0.5	0.5	0.5	0.3	0.3	0.3
L-lysine chloride (98% TCL)	4	2.8	2.8	2.8	2.6	2.6	2.6
Total	100	100	100	100	100	100	100

^1^ A 1 kg amount of vitamin–mineral premix provided 10,000 IU retinol, 2500 IU D_3_, 20 mg α-tocopherol, 0.5 mg thiamine, 5.0 mg riboflavin, 20.0 mg niacinamide, 1.0 mg pyridoxine, 0.02 mg cobalamin, 0.5 mg folic acid, 7.0 mg pantothenic acid, 2.5 mg menadione, 300 mg choline chloride, 45 mg Fe, 60 mg Mg, 50 mg Zn, 0.25 mg Se, and 1.3 mg I. IM—insect meal.

**Table 2:** Basic nutrients and amino acid composition of the insect meal and pheasant diets (g/kg).

Nutrients	Insect Meal (IM)	0–4 Weeks	5–8 Weeks			9–16 Weeks		
			Control	10% IM	20% IM	Control	10% IM	20% IM
Dry matter	978.6	878.8	877.2	881.2	884.5	877.3	880.4	882.3
AMEn, MJ kg^−2 1^	11.82	10.89	10.67	10.69	10.72	11.29	11.31	11.34
Crude fat	110.6	52.4	50.3	51.3	52.1	55.6	55.8	55.9
Crude ash	119.7	39.5	37.4	37.7	37.9	37.1	37.5	37.4
Calcium	9.42	11.41	9.52	9.6	9.61	8.73	8.67	8.69
Total phosphorus	8.51	7.52	6.99	6.88	6.92	6.87	6.74	6.85
Crude protein	518.8	265.1	239.6	242.5	244.2	203.7	204.8	205.6
Arginine	21.7	8.42	7.68	7.72	7.74	6.58	6.61	6.62
Histidine	12.4	10.43	9.73	9.75	9.78	8.43	8.44	8.52
Isoleucine	18.4	10.83	9.54	9.56	9.62	8.75	8.81	8.83
Leucine	29.2	10.43	9.51	9.49	9.53	7.89	7.91	7.84
Lysine	24.6	15.8	14.7	14.8	14.9	8.9	8.5	8.6
Methionine + cysteine	11.2	10.72	9.33	9.41	9.42	6.71	6.72	6.75
Phenylalanine	21.8	15.8	14.6	14.6	14.7	12.1	12.2	12.2
Threonine	16.8	11.2	8.7	8.8	8.7	8.5	8.6	8.6
Tryptophan	3.24	2.62	2.31	2.32	2.32	2.24	2.21	2.22
Valine	25.3	11.4	10.3	10.2	10.3	9.21	9.22	9.23

^1^ AMEn—metabolizable energy at zero nitrogen balance was calculated by the Fisher and McNab (1987) equations. IM—insect meal.

**Table 3:** Basic nutrients and amino acid composition of the insect meal and pheasant diets.

Item	Feeding Groups (FG)			Sex		*p* Value		
	Control	10% IM	20% IM	Male	Female	FG	Sex	FG × Sex
Body weight at 29th day of life, g	190	191	190	205	176	0.895	0.001	0.672
Body weight at slaughter, g	1094	1162	1231	1363	964	0.078	0.001	0.037
Average daily gains, g	10.76	11.56	12.39	13.78	9.38	0.069	0.001	0.041
Daily feed intake (29–112 d), g	57.7	57.9	57.8	59.9	55.7	0.645	0.058	0.093
Feed conversion ratio, g/g	5.36	5.01	4.67	4.35	5.94	0.032	0.001	0.042
Carcass weight, kg	0.72	0.76	0.83	0.89	0.65	0.082	0.001	0.035
Breast weight, g	224.2	225.3	226.5	258.4	191.6	0.347	0.001	0.112
Thigh weight, g	99.2	102.3	106.5	117.5	87.8	0.138	0.001	0.163

IM—insect meal. FG—feeding group.

**Table 4:** Nutrient content (%) in pheasant muscles.

Nutrients	Muscles	Feeding Groups (FG)			Sex		*p* Value		
		Control	10% IM	20% IM	Male	Female	FG	Sex	FG × Sex
Water	Breast	73.25	73.12	73.02	73.17	73.09	0.542	0.614	0.489
	Thigh	72.74	72.29	72.35	72.98	72.62	0.524	0.498	0.396
Protein	Breast	24.52	24.63	24.74	24.83	24.55	0.325	0.148	0.204
	Thigh	22.58	22.61	22.89	22.81	22.58	0.167	0.152	0.231
Lipids	Breast	0.49	0.52	0.55	0.45 ^a^	0.59 ^b^	0.175	0.036	0.098
	Thigh	2.56	2.61	2.72	2.51	2.75	0.189	0.058	0.136
Ash	Breast	1.19	1.18	1.21	1.23	1.17	0.512	0.286	0.193
	Thigh	1.33	1.34	1.37	1.39	1.31	0.421	0.502	0.187

^a,b^ the variables marked with different letters differ significantly at *p* < 0.05. IM—insect meal. FG—feeding group.

**Table 5:** Amino acid profile (mg/g) in pheasant muscles.

Amino Acids	Muscles	Feeding Groups (FG)			Sex		*p* Value		
		Control	10% IM	20% IM	Male	Female	FG	Sex	FG × Sex
Met	Breast	6.34	6.38	6.5	6.39	6.41	0.0041 *	0.5855	0.7588
	Thigh	5.37	5.59	5.71	5.55	5.56	0.0001 *	0.8024	0.674
Thr	Breast	10.54	10.55	10.62	10.59	10.55	0.7112	0.6438	0.8442
	Thigh	8.97	8.99	9.02	8.94	9.04	0.8433	0.1183	0.7633
Val	Breast	11.28	11.38	11.48	11.37	11.38	0.3329	0.9052	0.9389
	Thigh	9.11	9.05	9.1	9.04	9.13	0.6942	0.1954	0.6986
Ile	Breast	10.79	10.68	10.28	10.58	10.58	0.0045 *	0.9426	0.9333
	Thigh	9.12	9.03	8.99	9.03	9.06	0.3844	0.7134	0.9785
Leu	Breast	20.43	19.93	19.65	20.09	19.91	0.0067 *	0.3099	0.8003
	Thigh	17.4	17.24	17.17	17.28	17.26	0.183	0.895	0.9569
Phe	Breast	9.83	9.78	9.63	9.72	9.78	0.2027	0.4925	0.5689
	Thigh	8.57	8.45	8.36	8.43	8.49	0.2161	0.5166	0.8406
His	Breast	14.29	14.23	14.14	14.15	14.28	0.5766	0.2633	0.9063
	Thigh	7.69	7.75	7.85	7.76	7.77	0.528	0.9583	0.9662
Lys	Breast	20.1	20.2	20.28	20.06	20.33	0.5702	0.0605	0.6516
	Thigh	17.48	17.59	17.68	17.55	17.61	0.119	0.4272	0.9912
Asp	Breast	22.86	22.75	22.54	22.5	22.93	0.4578	0.0547	0.6856
	Thigh	19.56	19.46	19.45	19.4	19.58	0.5736	0.067	0.9922
Ser	Breast	8.89	8.89	8.8	8.83	8.89	0.6247	0.4995	0.6477
	Thigh	7.92	7.88	7.86	7.83	7.94	0.7033	0.0774	0.801
Glu	Breast	34.84	34.83	34.6	34.6	34.91	0.6794	0.2253	0.4816
	Thigh	31.28	31.41	31.41	31.08	31.65	0.797	0.0083 *	0.9219
Pro	Breast	6.78	6.72	6.75	6.69	6.82	0.9361	0.3348	0.9788
	Thigh	8.07	8.04	8.1	7.83	8.31	0.9353	0.0023 *	0.5228
Gly	Breast	9.22	9.18	9.15	9.11	9.26	0.7309	0.0528	0.4502
	Thigh	7.99	7.9	8.02	7.92	8.02	0.6782	0.368	0.3453
Ala	Breast	13.43	13.4	13.43	13.35	13.48	0.9824	0.3006	0.9317
	Thigh	11.36	11.35	11.58	11.42	11.44	0.3704	0.8627	0.9309
Arg	Breast	14.76	14.7	14.85	14.84	14.7	0.6204	0.2692	0.9434
	Thigh	12.54	12.5	12.71	12.54	12.62	0.4396	0.5747	0.9195
Tyr	Breast	10.64	10.59	10.63	10.67	10.57	0.8946	0.2799	0.7189
	Thigh	8.15	7.94	7.78	7.93	7.98	0.0194 *	0.555	0.1864

* statistically significant at *p* ≤ 0.01. IM—insect meal. FG—feeding group.

**Table 6:** Micro- and macronutrients (mg/kg) in pheasant muscles.

Minerals	Muscles	Feeding Groups (FG)			Sex		*p* Value		
		Control	10% IM	20% IM	Male	Female	FG	Sex	FG × Sex
Ca, g	Breast	0.24	0.25	0.25	0.23	0.26	0.3413	0.0026 *	0.8813
	Thigh	0.27	0.28	0.28	0.27	0.28	0.101	0.0222 *	0.7908
P, g	Breast	1.11	1.11	1.1	1.13	1.08	0.8884	0.0005 *	0.9344
	Thigh	1.05	1.06	1.07	1.09	1.03	0.6686	0.0001 *	0.7058
Mg, g	Breast	13.73	13.82	13.7	13.92	13.58	0.6323	0.0033 *	0.7812
	Thigh	13.01	13.06	13.12	13.17	12.96	0.3294	0.0022 *	0.8724
K, g	Breast	1.72	1.77	1.99	1.77	1.88	0.0001 *	0.0007 *	0.5452
	Thigh	2.32	2.34	2.37	2.3	2.38	0.2445	0.0036 *	0.9162
Na, g	Breast	3.17	3.19	3.22	3.22	3.16	0.3922	0.0517	0.9884
	Thigh	2.99	3.04	3.06	3.08	2.97	0.11	0.0007 *	0.8666
Fe, mg	Breast	78.69	79.55	81.21	77.55	82.08	0.1836	0.0006 *	0.839
	Thigh	105.35	106.38	108.18	99.92	113.35	0.011 *	0.0001 *	0.8778
Zn, mg	Breast	41.1	42.66	43.03	40.43	44.11	0.1035	0.0001 *	0.9700
	Thigh	74.83	75.84	76.19	72.96	78.28	0.0363 *	0.0001 *	0.7487
Cu, mg	Breast	7.95	8.09	8.19	8.02	8.13	0.0268 *	0.1047	0.0739
	Thigh	10.1	10.19	10.21	10.11	10.22	0.4732	0.1687	0.9040
Mn, mg	Breast	0.82	0.79	0.78	0.81	0.78	0.1182	0.0648	0.8890
	Thigh	0.92	0.92	0.9	0.92	0.91	0.1499	0.2073	0.9388

* statistically significant at *p* ≤ 0.01. IM—insect meal. FG—feeding group.

## Data Availability

The datasets generated during and/or analysed during the current study are available from the corresponding author upon reasonable request.
